# Risk factors for admission and length of stay in hospital for children with and without congenital anomalies: A EUROlinkCAT study

**DOI:** 10.23889/ijpds.v8i2.2297

**Published:** 2023-09-14

**Authors:** Joanne Given, Ester Garne, Joan Morris, Maria Loane, EUROlinkCAT Working Group

**Affiliations:** 1 Ulster University, Belfast, United Kingdom; 2 Paediatric Department, Hospital Lillebaelt, Kolding, Denmark; 3 St George's University of London, London, United Kingdom

## Objectives

To explore risk factors for hospital admission, and length of stay (LOS) in hospital, among children with congenital anomalies (CAs) and reference children without CAs in Europe.

## Methods

A European population-based data-linkage cohort study was conducted including children with CAs (born 1995-2014) registered in seven EUROCAT CA registries and children without CAs (reference children) living in the same geographical areas. Data on hospitalisation and LOS (1995-2015) for all children aged <1 year and 1-4 years were obtained by linkage to hospital discharge databases.

The effects of birth cohort, sex, gestational age, maternal age, multiple birth and socioeconomic status on risk of admission and LOS were estimated using Cox’s Proportional Hazards and negative binomial regression models. Random effects meta-analysis and quantile estimation methods were used to pool the estimates.

## Results

A total of 79,036 children with CAs and 2,016,042 reference children were linked to hospital records. Children with CAs born pre-term (<32 weeks) were more than twice as likely to be admitted (adj. HR 2.35, 95% CI 1.45-3.80) and had almost 8 times longer stays (adj. IRR 7.95, 95% CI 6.12-10.33) compared to children with CAs born at term. Reference children were almost six times as likely to be admitted (adj. HR 5.87, 95% CI 3.10-11.09), and had almost 50 times longer stays (adj. IRR 49.49, 30.92-79.21) compared to reference children born at term. Children with CAs and reference children born preterm were also at increased risk of admission at 1-4 years of age, although the effect was less than for children aged <1 year.

## Conclusion

The impact of risk factors for admission to hospital and LOS were similar between children with CAs and reference children but the impact was often greater in reference children. This study highlights the value of linking to hospital discharge records to obtain population-based information on morbidity for counselling parents.

